# Backward-Eulerian Footprint Modelling Based on the Adjoint Equation for Atmospheric and Urban-Terrain Dispersion

**DOI:** 10.1007/s10546-023-00807-z

**Published:** 2023-04-17

**Authors:** Hongyuan Jia, Hideki Kikumoto

**Affiliations:** Institute of Industrial Science, The University of Tokyo, 4-6-1 Komaba, Meguro-ku, Tokyo, 153-8505 Japan

**Keywords:** Adjoint equation, Footprint model, RANS simulation, Turbulent diffusion, Urban canopy

## Abstract

This study developed a backward-Eulerian footprint modelling method based on an adjoint equation for atmospheric boundary-layer flows. In the proposed method, the concentration footprint can be obtained directly by numerical simulation with the adjoint equation, and the flux footprints can be estimated using the adjoint concentration based on the gradient diffusion hypothesis. We first tested the proposed method by estimating the footprints for an ideal three-dimensional boundary layer with different atmospheric stability conditions based on the Monin–Obukhov profiles. It was indicated that the results were similar to the FFP method (Kljun et al. in Boundary-Layer Meteorol 112:503–523, 2004, 10.1023/B:BOUN.0000030653.71031.96; Geosci Model Dev 8:3695–3713, 2015, 10.5194/gmd-8-3695-2015) for convective conditions and the K–M method (Kormann and Meixner in Boundary-Layer Meteorol 99:207–224, 2001, 10.1023/A:1018991015119) for stable conditions. The proposed method was then coupled with the Reynolds averaged Navier–Stokes model to calculate the footprints for a block-arrayed urban canopy. The results were qualitatively compared to the results from the Lagrangian-Large-Eddy-Simulation (LL) method (Hellsten et al. in Boundary-Layer Meteorol 157:191–217, 2015, 10.1007/s10546-015-0062-4). It was shown that the proposed method reproduced the main features of footprints for different sensor positions and measurement heights. However, it is necessary to simulate the adjoint equation with a more sophisticated turbulence model in the future to better capture turbulent effects in the footprint modelling.

## Introduction

The development of the eddy covariance (EC) technique (Aubinet et al. 2012) has enabled the monitoring of mass and energy exchange in the atmospheric boundary layer (ABL). In addition to the concentration of the target gas, the vertical flux can be calculated by combining the wind velocity measured by the sensors. However, the obtained concentration or flux is the integrated product of all potential sources in the upwind area; therefore, it is necessary to interpret the measurements to extract detailed information about the sources. Schuepp et al. (1990) proposed the “footprint” concept to connect the measurements with the sources.

The footprint function describes the relative contribution of each elemental source area upstream of the sensor to the measured concentration or vertical flux. Previously, the interpretation of measurements using the footprint function has been employed in different monitoring scenarios in urban areas and the results have been encouraging. For example, Sugawara et al. (2021) analyzed the changes in anthropogenic CO_2_ emissions in the urban area of Tokyo during the COVID-19 state of emergency. Ando and Ueyama (2017) identified the surface energy exchange in a dense urban area in Osaka, Japan, based on two-year EC measurements and footprints. Furthermore, Lauvaux et al. (2016) estimated CO_2_ source distributions over the city of Indianapolis based on tower network observations, footprint modelling, and the Bayesian inversion method. Baldocchi et al. (2001) stated that the FLUXNET network and appropriate footprint knowledge can help elucidate regional and global ecosystem exchanges.

In addition to explaining the measurements, the footprint model can also improve monitoring quality. Footprint predictions can serve as guidance for the planning of sensor networks and the design of sampling characteristics (Leclerc et al. 2003). Levin et al. (2020) designed a dedicated flask-sampling strategy for integrated carbon observation system stations based on footprint information.

To ensure the efficiency of the above applications, it is critical to accurately and quickly estimate the footprint function for each sensor. Since the proposal of the footprint concept, several modelling methods have been developed. There are three main types of modelling methods. At an early stage, researchers developed an analytical model for the footprint based on the advection–diffusion equation. Several approximation solutions have been derived for situations with thermally neutral stratification and a constant velocity profile (Schuepp et al. 1990; Horst and Weil 1992; Hsieh et al. 2000). Kormann and Meixner (2001) introduced the Monin–Obukhov profiles into the analytical analysis (K–M model) to represent the effects of different boundary layer stabilities, which are still commonly used in modern applications. Although these analytical models are cost-effective and user-friendly, they can only deal with a horizontal homogeneous turbulent field and lose validity outside of the surface layer (Kljun et al. 2002).

The second type of footprint model is the Lagrangian stochastic approach. Based on the Langevin equation, the trajectory of each scalar particle emitted from the source area is calculated (forward mode), or the trajectory of each scalar particle released from the sensor is tracked backward in time with the inverse flow (backward mode) (Vesala et al. 2008). A representative Lagrangian footprint model was developed by Kljun et al. (2002), who considered various ABL stratification conditions. This model was then parameterized by an approximation formula and evolved into the rapid Flux Footprint Predictions (FFP) model (Kljun et al. 2004, 2015). However, in these models, the turbulence influence is commonly represented by a one-point probability density function (PDF) of the Eulerian velocity field to reduce calculation costs. This predefined turbulent field requires revisions when the target location is changed. It is also difficult to properly describe heterogeneous turbulent fields using only one velocity PDF. With the development of computational techniques and the growing need to handle complex surface conditions, such as urban built environments, large-eddy simulation (LES) has replaced PDF velocity to directly simulate the turbulent flow field, which then drives the transport of scalar particles. Several studies have applied the LES + Lagrangian (LL) method to investigate the footprint in the ABL (Glazunov et al. 2016), urban canopy models (Hellsten et al. 2015), and real urban areas (Auvinen et al. 2017). One of the unavoidable weaknesses of these Lagrangian methods is the heavy computational burden caused by the use of many trajectories, which is indispensable to the stable convergence of the modelling.

The third type of footprint model is based on the Eulerian simulation of the Navier–Stokes equations. The workflow was presented by Sogachev et al. (2002) and Sogachev and Lloyd (2004), whereby the concentration field of each surface cell was repeatedly simulated and the flux footprint was then estimated based on a one-and-a-half order closure with concentration results. This approach may be able to handle footprint modelling over heterogeneous terrains; however, it must simulate the dispersion fields for all potential sources, which is burdensome and inefficient. These disadvantages may also be the reason that, except for this Eulerian model, other Eulerian methods are rare in the literature. Therefore, it is meaningful to develop a more effective Eulerian modelling method that is not hampered by a large number of particles or potential sources.

Recently, an adjoint concentration equation has attracted attention in source term estimation studies (Pudykiewicz 1998; Keats et al. 2007; Jia and Kikumoto 2021). It is a powerful tool for constructing the source–receptor relationship, which has a physical meaning similar to that of the concentration footprint. As an Eulerian dispersion simulation, the merit of the adjoint equation is that the contributions of all sources to the concentration measurement of a sensor are estimated from the view of the sensor by inverse simulation. Considering that the number of sensors is finite and that the dispersion is simulated by the transport equation of passive scalar rather than particle trajectories in the Eulerian method, the adjoint equation can save significant computational resources. Although the current usage of the adjoint equation mainly focuses on concentration estimation, the flux may be estimated from the concentration using the K-theory (Sogachev and Lloyd 2004). Therefore, a backward Eulerian footprint modelling approach could be established based on the adjoint equation.

In this study, we propose this type of backward Eulerian modelling method for footprints. Following the definition of footprints in previous studies (Schuepp et al. 1990; Kljun et al. 2002), the proposed method is concerned with the surface source in the target domain, which includes the whole bottom surface, to reflect the response of the sensor when possible sources appear anywhere. In numerical modelling, the surface source can be divided into ‘point’ sources by the grid. Hence, we derived the proposed method based on the point sources as shown in Sect. 2. This derivation process enables the proposed method to deal with the spatially continuous surface sources, as well as the discrete point sources sparsely distributed in the domain.

The simulation of the adjoint equation requires a turbulent flow field in the target domain. Therefore, the three-dimensional wind velocity field is a prerequisite, which can be simulated by computational fluid dynamics (CFD) with appropriate boundary conditions (Blocken 2015). In other words, the wind direction and mean velocity profile are necessary information for the application of the proposed method. In reality, the wind direction and profile are unsteady, so the footprints are modelled for each period when the wind direction and profile are almost steady. In each period, because the target domain, wind flow field, and sensor are unchanged, the footprint function is steady and independent of the temporal releasing characteristics of the surface source. Additionally, since the footprint function is calculated inversely from the view of sensors by the adjoint equation, users do not require much information about the sources (such as the location and number of sources). This is one of the advantages of the proposed method.

For the remaining content, the definition of the footprint function and a detailed introduction to the proposed method are presented in Sect. 2. Section 3 describes the use of the proposed method to model the footprint of a three-dimensional atmospheric boundary layer with different thermal stabilities. The robustness of the adjoint relationship in the backward flux estimation was first evaluated, and then comparisons of our results with the analytical solutions (K–M model) and Lagrangian results (FFP model) are discussed. Section 4 provides an application of the proposed method to footprint modelling in a block-arrayed urban canopy model. The footprints obtained were qualitatively compared to those simulated using the LL method. Section 5 provides concluding remarks.

## Methodology

### Definition of the Footprint Function

The concept of the footprint function can be expressed by the following equation:

1 $$\eta \left({{\varvec{x}}}_{\mathrm{m}}\right)= \int_{\mathcal{S}}^{{}{ }}f\left({{\varvec{x}}}_{\mathrm{m}}|{\varvec{x}}\right){q}_{s}\left({\varvec{x}}\right)d{\varvec{x}},$$

This integration is conducted on the two-dimensional bottom surface $$\mathcal{S}$$ of the target domain.　$${\varvec{x}}$$ is the coordinate on$$\mathcal{S}$$, which represents the locations of all potential sources or sinks. $${{\varvec{x}}}_{\mathrm{m}}$$ represents the coordinate of the target sensor$$,$$ and $$\eta $$ is the measured quantity. When $$\eta
$$ is the concentration ($$\mathrm{g}\,
{\mathrm{m}}^{-3}$$) or vertical flux ($$\mathrm{g}\,{\mathrm{m}}^{-2}
\,{\mathrm{s}}^{-1}$$) of the gas, $$f\left({{\varvec{x}}}_{\mathrm{m}}|{\varvec{x}}\right)$$ is the concentration ($$\mathrm{s}\,
{\mathrm{m}}^{-3}$$) or flux footprint function ($${\mathrm{m}}^{-2}$$) between sensor $${{\varvec{x}}}_{\mathrm{m}}$$ and all potential sources on$$\mathcal{S}$$. $${q}_{s}$$ denotes the strength ($$\mathrm{g}\,{\mathrm{m}}^{-2}
\,{\mathrm{s}}^{-1}$$) of the source or sink. Notice that the unit set is based on the mass concentration, and the unit set for the volume concentration can be written similarly.

In the integration, it is not necessary that ${q}_{s}$ is spatially continuous because there may be only discrete sources in the target domain. In such a case, ${q}_{s}\left({\varvec{x}}\right)$ is a set of delta-spikes and the value is 0 at the location where there is no source. If the footprint function and source strength distribution are known, the resultant measurements can be integrated quickly. In contrast, if the measurements and footprint functions are available, it is possible to inversely estimate the information of the sources that caused these measurements.

### Eulerian Modelling Approach

Footprint modelling requires a dispersion simulation based on preparatory turbulent flow fields, which can be simulated by the Navier–Stokes equation and the continuity equation in the Eulerian approach. There are many mature turbulence modelling approaches, such as the Reynolds-averaged Navier–Stokes (RANS) method, LES, and Direct Numerical Simulation (DNS). The evolution of the concentration field $C$ ($\mathrm{g}\, {\mathrm{m}}^{-3}$) of the passive scalar resulting from the strength function ${q}_{s}$ can be written as:

2a $$\frac{\partial C}{\partial t}+\left({\varvec{u}}\cdot \nabla \right)C-\nabla \cdot \left(K\nabla C\right)={q}_{s}\left({\varvec{x}}\right),$$

2b $${\nabla }_{{\varvec{n}}}C
=0\,\,\mathrm{ at }\,\,\partial\Omega ,$$

2c $$C\left({\varvec{x}}, t=0\right)=0.$$

This equation has a spatial domain $$\Omega
$$ and a time range of $$[0,T]$$. $$K$$ is the mass diffusivity, and $${\nabla
}_{{\varvec{n}}}$$ is a directional derivative normal to the boundary. Please note that only an ideal format of the transport equation for passive scalar is given here, without considering the chemical reaction, condensation, radiation, and other properties of specific scalars.

Based on the physical meaning of Eq. (1), it is considered that there is only an infinitely small surface source located at ${{\varvec{x}}}_{\mathrm{s}}$ in the target domain. It releases pollutants at a rate of ${Q}_{0}$ ($\mathrm{g}\cdot {\mathrm{s}}^{-1}$), which means that the strength distribution is:

3 $${q}_{s}\left({\varvec{x}}\right)={Q}_{0}\delta \left({\varvec{x}}-{{\varvec{x}}}_{s}\right).$$

Here, $\delta (\cdot)$ is a two-dimensional Dirac delta function with the unit of ${m}^{-2}$. Thus, Eq. (1) changes to:

4 $$\eta \left({{\varvec{x}}}_{\mathrm{m}}\right)= \int_{{\varvec{x}}}^{{}{ }}f\left({{\varvec{x}}}_{\mathrm{m}}|{{\varvec{x}}}_{\mathrm{s}}\right){Q}_{0}\delta \left({\varvec{x}}-{{\varvec{x}}}_{s}\right)d{\varvec{x}}={Q}_{0}f\left({{\varvec{x}}}_{\mathrm{m}}|{{\varvec{x}}}_{\mathrm{s}}\right).$$

Then, the footprint function can be calculated by:

5 $$f\left({{\varvec{x}}}_{\mathrm{m}}|{{\varvec{x}}}_{\mathrm{s}}\right)=\frac{\eta
\left({{\varvec{x}}}_{\mathrm{m}}\right)}{{Q}_{0}}.$$

When $${Q}_{0}=1(\mathrm{g}\cdot
{\mathrm{s}}^{-1})$$, the resulting concentration or flux at the sensor equals the footprint function value even though their units are different. When the measurement $$\eta
\left({{\varvec{x}}}_{\mathrm{m}}\right)$$ is the time-averaged concentration $$\overline{C\left({{\varvec{x}}}_{\mathrm{m}}|{{\varvec{x}}}_{s}\right)}$$ ($$\mathrm{g}\cdot
{\mathrm{m}}^{-3}$$), the concentration footprint $${f}_{\mathrm{c}}\left({{\varvec{x}}}_{\mathrm{m}}|{{\varvec{x}}}_{s}\right)$$ ($$\mathrm{s}\cdot
{\mathrm{m}}^{-3}$$) can be calculated by:

6 $${f}_{c}\left({{\varvec{x}}}_{m}|{{\varvec{x}}}_{s}\right)=\frac{\overline{C\left({{\varvec{x}}}_{m}|{{\varvec{x}}}_{s}\right)}}{1\mathrm{
g}\cdot {\mathrm{s}}^{-1}}.$$

**Fig. 1:**
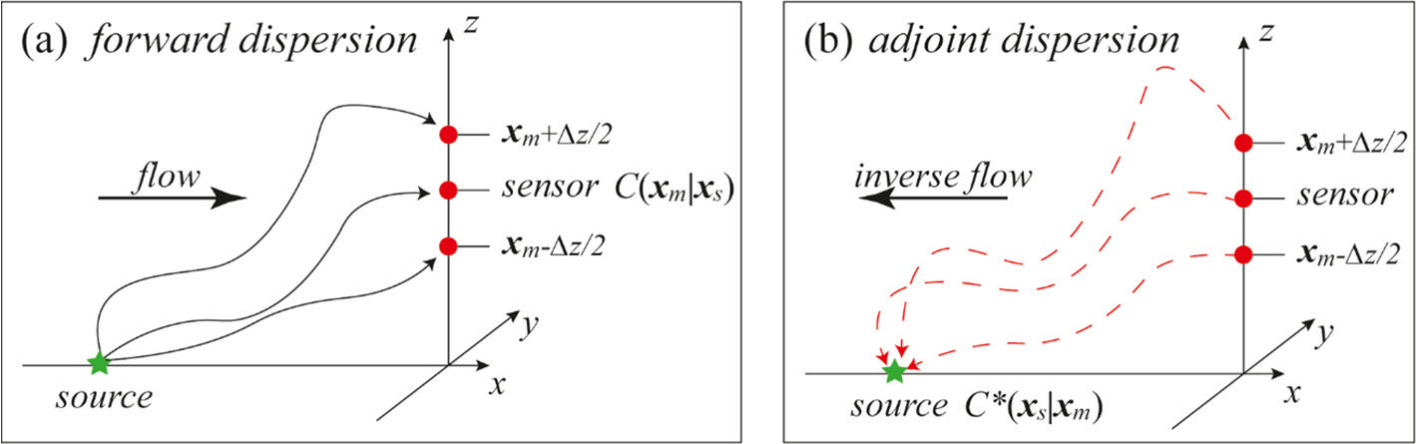
Schematic of forward dispersion and adjoint dispersion

As for the flux footprint ${f}_{\mathrm{f}}$, the mean turbulent flux ${f}_{\mathrm{tf}}$ at sensor ${{\varvec{x}}}_{\mathrm{m}}$ caused by source ${{\varvec{x}}}_{s}$ is estimated by the mean concentration using the K-theory (Sogachev and Lloyd 2004):

7a $${f}_{\mathrm{f}}\left({{\varvec{x}}}_{\mathrm{m}}|{{\varvec{x}}}_{s}\right)\boldsymbol{ }=\boldsymbol{ }\overline{{u }_{z}({{\varvec{x}}}_{\mathrm{m}})}\cdot \overline{C\left({{\varvec{x}}}_{\mathrm{m}}|{{\varvec{x}}}_{s}\right)}+{f}_{\mathrm{tf}}\left({{\varvec{x}}}_{\mathrm{m}}|{{\varvec{x}}}_{s}\right),$$

7b $${f}_{\mathrm{tf}}\left({{\varvec{x}}}_{\mathrm{m}}|{{\varvec{x}}}_{s}\right) = \overline{{u}{'}_{z}({{\varvec{x}}}_{\mathrm{m}})C'({{\varvec{x}}}_{\mathrm{m}}|{{\varvec{x}}}_{s})} \approx -{K}_{t}\left({{\varvec{x}}}_{\mathrm{m}}\right){\left.\frac{d\overline{C}}{dz }\right|}_{{{\varvec{x}}}_{\mathrm{m}}},$$

where ${K}_{t}$ is the local eddy-diffusion coefficient, $\boldsymbol{^{\prime}}$ denotes the temporal fluctuation of a quantity, and $\stackrel{-}{}$ is the time-averaging operator. This relationship is widely used in steady-state dispersion simulations to model turbulent flux and is also known as the gradient diffusion hypothesis (Combest et al. 2011). The gradient of the mean concentration can be evaluated through the concentrations of the adjacent cells upward and downward from the sensor (Sogachev and Lloyd 2004) as shown in Fig. 1(a), which means:

8 $${\left.\frac{d\overline{C}}{dz
}\right|}_{{{\varvec{x}}}_{\mathrm{m}}} \approx
\frac{\overline{C\left[\left({{\varvec{x}}}_{\mathrm{m}}+\frac{\Delta
z}{2}\right)|{{\varvec{x}}}_{s}\right]}-\overline{C\left[\left({{\varvec{x}}}_{\mathrm{m}}-\frac{\Delta
z}{2}\right)|{{\varvec{x}}}_{s}\right]}}{\Delta
z}.$$

### Proposed Backward Method with the Adjoint Equation

One problem with the above method is that the concentration fields of all sources must be simulated, which requires a large amount of computational resources for real applications. This study proposes the use of the adjoint equation to mitigate this problem.

If we define a linear operator ${\varvec{L}}(\cdot)$ in a Hilbert space such that:

9 $${\varvec{L}}(\cdot)\equiv \boldsymbol{ }\frac{\partial (\cdot)}{\partial t}+\left({\varvec{u}}\cdot \nabla \right)\left(\cdot \right)-\nabla \cdot \left(K\nabla \left(\cdot \right)\right),$$

then the dispersion from a point source can be represented by the transformation of function $C$ by ${\varvec{L}}$:

10 $${\varvec{L}}\left(C\right) = {Q}_{0}\delta \left({\varvec{x}}-{{\varvec{x}}}_{s}\right).$$

Due to the Lagrange duality relationship (Christensen 2010, pp. 70–74), the adjoint operator ${{\varvec{L}}}^{\boldsymbol{*}}$ corresponds to ${\varvec{L}}$ exists, which is:

11 $$\langle {C}^{*}{\varvec{L}}\left(C\right)\rangle  = \langle {{\varvec{L}}}^{\boldsymbol{*}}\left({C}^{*}\right)C\rangle ,$$

where ${C}^{*}$ is the adjoint concentration field and $\langle \cdot \rangle$ is the linear product operator defined in the same Hilbert space as:

12 $$\langle {C}^{*}{\varvec{L}}\left(C\right)\rangle  = {\int }_{0}^{T}dt{\int }_{\Omega }({C}^{*}\cdot {\varvec{L}}\left(C\right))d\Omega .$$

According to previous studies (Marchuk 1995), ${{\varvec{L}}}^{\boldsymbol{*}}$ can be expressed as:

13 $${{\varvec{L}}}^{{*}}(\cdot)\equiv -\frac{\partial (\cdot)}{\partial t}-\left({\varvec{u}}\cdot \nabla \right)(\cdot)-\nabla \cdot \left(K\nabla (\cdot)\right).$$

It is interesting to note that the ${{\varvec{L}}}^{\boldsymbol{*}}\left({C}^{*}\right)$ can be regarded as the transport process of a hypothetical tracer emitted from sensor ${{\varvec{x}}}_{m}$ with the rate of ${Q}_{0}$, which means:

14a $${{\varvec{L}}}^{\boldsymbol{*}}\left({C}^{*}\right) = {Q}_{0}\delta \left({\varvec{x}}-{{\varvec{x}}}_{m}\right),$$

14b $${\nabla }_{{\varvec{n}}}{C}^{*} = 0\,\,\mathrm{ at }\,\,\partial\Omega ,$$

14c $${C}^{*}\left({\varvec{x}}, t = T\right) = 0.$$

In this dispersion process, the tracers are transported by the inverse flow $-{\varvec{u}}({\varvec{x}}, t)$ from time $T$ to time 0 with a negative time-step increment. If we incorporate Eqs. (10) and (14) into Eq. (11), the adjoint relationship can be obtained:

15 $${Q}_{0}\overline{{C }^{*}\left({{\varvec{x}}}_{s}|{{\varvec{x}}}_{m}\right)}\boldsymbol{ }=\boldsymbol{ }{Q}_{0}\overline{C\left({{\varvec{x}}}_{\mathrm{m}}|{{\varvec{x}}}_{s}\right)}.$$

In this case, Eq. (15) indicates that the time-averaged concentration of the target gas at the sensor $\overline{C\left({{\varvec{x}}}_{\mathrm{m}}|{{\varvec{x}}}_{s}\right)}$ equals the time-averaged concentration of the adjoint tracer at the source $\overline{{C }^{*}\left({{\varvec{x}}}_{s}|{{\varvec{x}}}_{m}\right)}$. Therefore, the dispersion equations do not need to be solved for all potential sources. Instead, only a few adjoint equations for the sensors are sufficient, which reduces the calculation requirements considerably.

According to this adjoint relationship, the forward concentration can be equally replaced by the adjoint concentration in Eq. (6); therefore:

16 $${f}_{c}\left({{\varvec{x}}}_{m}|{{\varvec{x}}}_{s}\right)=\frac{\overline{{C }^{*}\left({{\varvec{x}}}_{s}|{{\varvec{x}}}_{m}\right)}}{1 \mathrm{g}\cdot {\mathrm{s}}^{-1}}.$$

Then, the turbulent flux value at the sensor can be similarly transferred based on Eqs. (7) and (8), and the concentration gradient can be approximated:

17a $${f}_{\mathrm{tf}}\left({{\varvec{x}}}_{\mathrm{m}}|{{\varvec{x}}}_{s}\right) = -{K}_{t}\left({{\varvec{x}}}_{\mathrm{m}}\right){\left.\frac{d\overline{C}}{dz }\right|}_{{{\varvec{x}}}_{\mathrm{m}}},$$

17b $${\left.\frac{d\overline{C}}{dz }\right|}_{{{\varvec{x}}}_{\mathrm{m}}} \approx \frac{\overline{C\left[\left({{\varvec{x}}}_{\mathrm{m}}+\frac{\Delta z}{2}\right)|{{\varvec{x}}}_{s}\right]}-\overline{C\left[\left({{\varvec{x}}}_{\mathrm{m}}-\frac{\Delta z}{2}\right)|{{\varvec{x}}}_{s}\right]}}{\Delta z}$$

17c $$\qquad\qquad\qquad= \frac{\overline{{C }^{*}\left[{{\varvec{x}}}_{s}|\left({{\varvec{x}}}_{\mathrm{m}}+\frac{\Delta z}{2}\right)\right]}-\overline{{C }^{*}\left[{{\varvec{x}}}_{s}|\left({{\varvec{x}}}_{\mathrm{m}}-\frac{\Delta z}{2}\right)\right]}}{\Delta z}.$$

Note that the adjoint concentration fields in Eq. (17) are caused by releases from the adjacent cells with coordinates of $\left({{\varvec{x}}}_{\mathrm{m}}+\frac{\Delta z}{2}\right)$ and $\left({{\varvec{x}}}_{\mathrm{m}}-\frac{\Delta z}{2}\right),$ as shown in Fig. 1(b), as opposed to the sensor cell ${{\varvec{x}}}_{\mathrm{m}}$. Therefore, when the footprint of one sensor is modeled, three adjoint equations must be simulated, which is still significantly fewer than the total potential sources.

## Flux Simulation for the Atmospheric Boundary Layer

The first application of the proposed method is to estimate the flux footprint in an ideal three-dimensional ABL. In large-scale modelling of the footprint, different terrains and atmospheric stabilities can be approximated by a boundary layer with an appropriate adjustment of coefficients. Therefore, since Kormann and Meixner (2001) provided an analytical solution based on this scenario, the following research (Kljun et al. 2004; Wang et al. 2018) tested their methods using this case. In this section, the proposed method is used to estimate flux footprints in the ABL. The results are compared to those of the analytical K–M method (Kormann and Meixner 2001) and the FFP model based on the Lagrangian stochastic tracer method (Kljun et al. 2002, 2004).

### Simulation Settings

Seven atmospheric stability conditions are considered, as shown in Table 1. For footprint modelling, a boundary layer size of $4000 \,\,\mathrm{m}\left(x\right) \times 600 \,\,\mathrm{m}\left(y\right) \times 200 \,\,\mathrm{m}(z)$ is set as the calculation domain. Forward and adjoint dispersions are simulated using Eqs. (2) and (10) with the finite volume method using OpenFOAM v2112 (Weller et al. 1998). The advection scheme for dispersion is set as a first-order bounded upwind. As for the mesh setting, in the horizontal plane, a uniform hexahedral orthogonal grid with $5\,\,\mathrm{ m}\left(x\right) \times 5 \,\,\mathrm{m}(y)$ is utilized for the entire domain. To accurately simulate the dispersion near the bottom wall, a grid edge of $1 \mathrm{m}(z)$ was imposed from 0 to 50 m in the vertical direction and then expanded at a ratio of 1.08 to 200 m.

**Table 1:** Seven cases with stabilities defined by coefficients

Stability	Friction speed (m s^−1^)	Obukhov length (m)	Roughness height (m)	Sensor height (m)
Strongly convective	0.2	−5	0.01	10
Forced convective	0.2	−30	0.01	7.5
Slightly convective	0.3	−650	0.01	90
Neutral	0.5	$\infty$	0.01	75
Slightly stable	0.4	1000	0.01	60
Stable	0.3	130	0.01	18.75
Strongly stable	0.3	84	0.01	15

Despite the use of CFD simulation, the dispersion processes are solved by OpenFOAM, but the streamwise velocity and turbulent diffusion coefficients are assigned to fixed vertical profiles, which are synthesized by the Monin–Obukhov profiles (Monin and Obukhov 1954):

18a $$\overline{{U }_{x}}\left(z\right) = \frac{{u}_{*}}{\kappa }\left[\mathrm{ln}\frac{z}{{z}_{0}}+{\psi }_{m}\left(\frac{z}{L}\right)\right],$$

18b $${K}_{t}\left(z\right) = \frac{\kappa {u}_{*}z}{{\varphi }_{c}(z/L)},$$

where $\kappa =0.4$ is the von Kármán constant. The boundary layer stratification condition is characterized by the friction speed ${u}_{*}$, Obukhov length $L$, and roughness height ${z}_{0}$, as listed in Table 1. The Businger–Dyer relationships describing the stability dependence of the profiles are (Paulson 1970; Dyer 1974):

19a $${\psi }_{m} =
\left\{\begin{array}{l}\frac{5z}{L} \,\,\text{for}\,\, 0<z/L<1\\
-2\mathrm{ln}\left[\frac{1+\xi
}{2}\right]-\mathrm{ln}\left[\frac{1+{\xi
}^{2}}{2}\right]+2\mathrm{arctan}\xi -\frac{\pi }{2}
for-2<z/L<0\end{array}\quad \text{with}\,\,\right.\xi =
{(1-16z/L)}^{1/4}\,\,
\mathrm{and}$$

19b $${\varphi }_{c} =
\left\{\begin{array}{l}1+\frac{5z}{L} \,\,\text{for}\,\, 0<z/L <1\\
{(1-16z/L)}^{-1/2} \,\,\text{for}\,\,
-2<z/L<0\end{array}\right.$$

### Verification of the Adjoint Equation in the Backward Flux Estimation

First, we verify the applicability of the backward concentration or flux estimation based on the adjoint equation. The forward dispersion of a point source and the adjoint dispersion of a point sensor are simulated to check whether the forward resultant concentration or flux and backward estimations are matched. Flux is estimated based on the concentration results from both the forward and backward simulations; therefore, it is only necessary to determine whether $\overline{{C }^{*}\left({{\varvec{x}}}_{s}|{{\varvec{x}}}_{m}\right)}$ is the same as $\overline{C\left({{\varvec{x}}}_{\mathrm{m}}|{{\varvec{x}}}_{s}\right)}$.

We present the results of the strongly convective case here because the verification processes for the different stability conditions are similar. For the verification, the sensor is placed at a height of 10 m, as shown Table 1, and the source is positioned on the bottom surface $(z=0.5 \mathrm{m})$ 150 m upstream of the sensor. The adjoint concentration fields released from three points $(z = 9.5, 10.0, \mathrm{and} 10.5 \mathrm{m}$) are calculated for the flux estimation, and $\Delta z$ in Eq. (13) was 1 m.

**Fig. 2:**
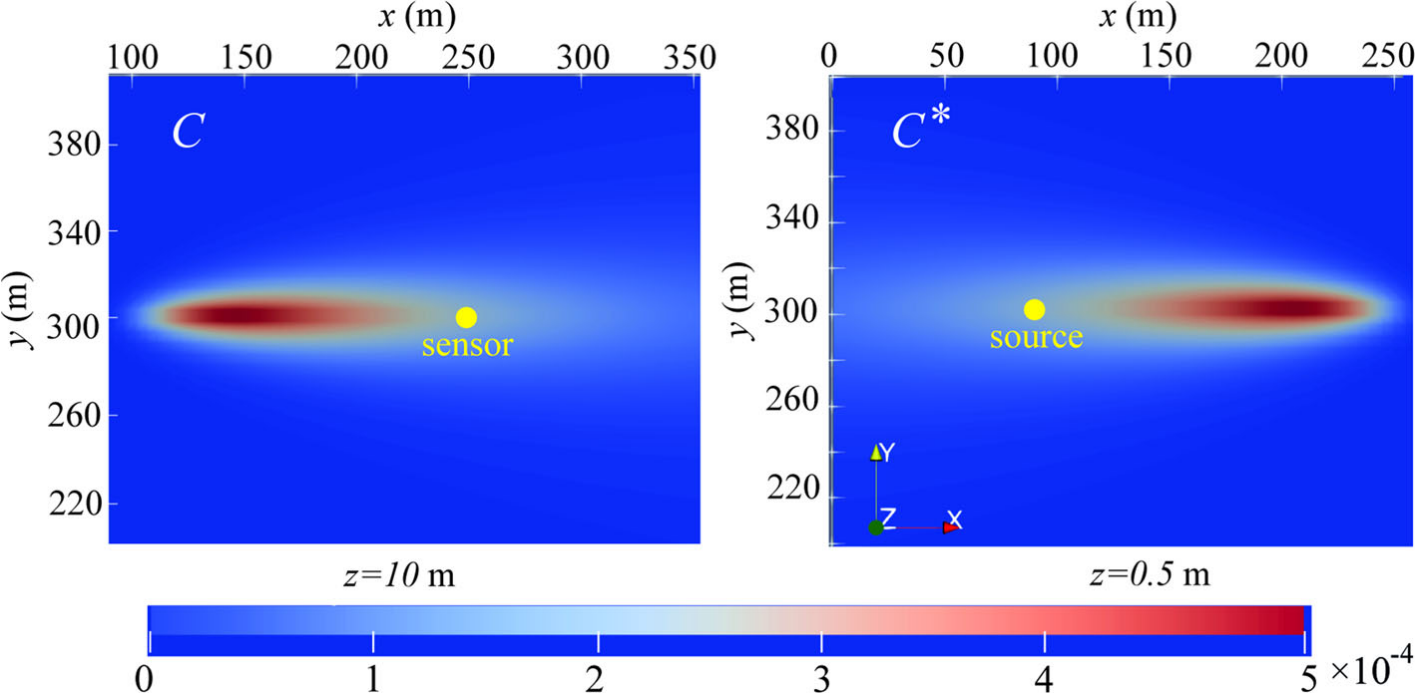
Concentration distribution of the source (left) and adjoint concentration distribution of the sensor (right)

Figure 2 shows the horizontal distributions of the forward-simulated concentration of the source and the adjoint concentration of the sensor. The adjoint concentration is dispersed upstream because it is driven by reverse flow. Although these planes have two heights, their distributions are analogous. The values of $\overline{C\left({{\varvec{x}}}_{\mathrm{m}}|{{\varvec{x}}}_{s}\right)}$ and $\overline{{C }^{*}\left({{\varvec{x}}}_{s}|{{\varvec{x}}}_{m}\right)}$ are extracted and their difference is shown in Table 2. It is confirmed that the concentration measured at each receptor is close to the adjoint concentration at the source from each receptor. The numerical error caused by backward estimation is imperceptible in the current case.

**Table 2:** Comparisons between the forward dispersion concentration and estimations based on the adjoint dispersion

Receptors	Quantities	
	$\overline{C\left({{\varvec{x}}}_{\mathrm{m}}|{{\varvec{x}}}_{s}\right)}$ in the forward simulation	Difference between $\overline{C\left({{\varvec{x}}}_{\mathrm{m}}|{{\varvec{x}}}_{s}\right)}$ and $\overline{{C }^{*}\left({{\varvec{x}}}_{s}|{{\varvec{x}}}_{m}\right)}$
${{\varvec{x}}}_{\mathrm{m}}-\frac{\Delta z}{2}$	2.26 × 10^−4^	2.63 × 10^−8^
${{\varvec{x}}}_{\mathrm{m}}$	2.05 × 10^−4^	2.71 × 10^−8^
${{\varvec{x}}}_{\mathrm{m}}+\frac{\Delta z}{2}$	2.04 × 10^−4^	2.74 × 10^−8^

However, deviations caused by backward estimation are unavoidable because the numerical scheme may perform differently in the forward and backward directions. Furthermore, the backward simulation may be biased by the spatiotemporal interpolation errors in unsteady and strongly curved confluent flow (Dahl et al. 2012). The backward parcel trajectories may be considerably different from the forward ones because of the errors caused by the temporally interpolated velocity field or the spatial interpolation in the strongly curved flow. Although this problem is analyzed in the Lagrangian backward simulation, analogous problems may occur in the proposed method when the unsteady adjoint equation is simulated based on the temporal interpolated velocity field or the strongly curved flow field. Before the potential risk brought by the backward simulation is clarified by future research, special attention is still required for complex turbulent flow fields.

### Simulation Results

**Fig. 3:**
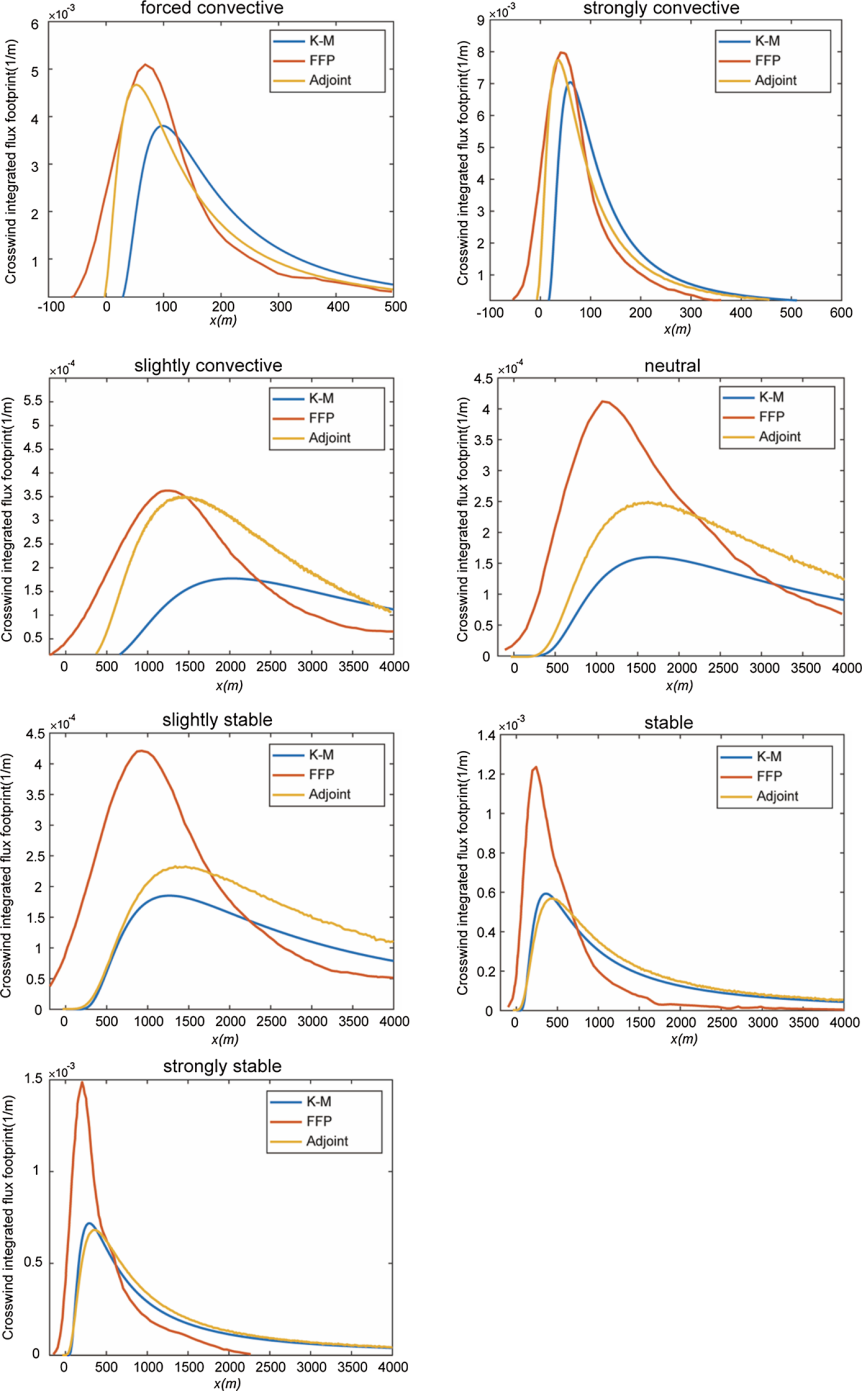
Comparisons of the crosswind integrated flux footprint modelling results from the K–M method, FFP method, and proposed adjoint method in the ABLs with different stability conditions

The crosswind integrated flux footprints for the three methods are shown in Fig. 3. In all three models, when the stability changes from convective to neutral, the streamwise extent of the footprint increases, and the peak value decreases. When the stability changes from neutral to stable, the extent of the footprint shrinks, and the peak value become larger again.

Under convective situations, where turbulent diffusion during dispersion has a dominant effect on the concentration and flux distribution, the proposed method yields results similar to that of the FFP model. The peak values and locations are almost identical. In comparison, K–M underestimates the peak value and overestimates the peak location and range extent. The estimation discrepancy is caused by the absence of turbulent diffusion in the horizontal direction in the K–M method (Kormann and Meixner 2001). Unlike the K–M method, the proposed method applies a homogeneous $K$ in all directions by the Monin–Obukhov profile, so the turbulent diffusion is better captured, and the results are closer to those of the FFP. However, it should be noted that the footprints upstream of the source do not appear in the proposed method, which may indicate that the synthesized diffusion coefficients are still insufficient to accurately capture the turbulent characteristics. A more comprehensive flow field can further improve the estimation performance.

As the ABL becomes increasingly stable, the turbulent diffusion of the passive scalar gradually attenuates. As a result, neglecting horizontal turbulent diffusion in the K–M does not generate a large difference when compared to the Monin–Obukhov profile used in the proposed method, and the two footprints grow closer. In contrast, the FFP method generates two times larger peak values, smaller peak locations, narrower footprint ranges, and non-zero footprints upstream of the source.

Overall, the proposed adjoint method performs similarly to the FFP method under convective conditions and comparably to the K–M method in terms of stable stability in this study case. One of the main reasons for this is probably because the turbulent fields used in the three methods are different. When the proposed method is compared to the K–M method, the main difference is that the horizontal turbulent diffusion is ignored in the latter. This can explain why the proposed method is similar to the K–M method in terms of stable stability, while it is different from the K–M method in terms of convective stability. It was also demonstrated that the absence of horizontal turbulence results in similar deviations between the K–M and FFP methods (Kljun et al. 2003). In theory, the FFP method should produce the best estimation because it considers the Reynolds stress, particle velocity variance, and vertical velocity skewness in the dispersion by the LPDM (Rotach et al. 1996) Lagrangian model. When the proposed method is compared to the FFP method, the two methods own close peak values and peak locations in the unstable stabilities indicates that the Monin–Obukhov profile and LPDM model similarly reflects the turbulent diffusion and advection. However, the peak values of FFP are much higher than those of the proposed method and K–M method in the stable stabilities. The possible reason is that the non-Gaussian crosswind dispersion and velocity skewness are considered in the LPDM model. It should be said that it may be difficult to analyze the reasons further because the specific parameters for turbulence in LPDM model are unavailable, which is a limitation of the current study.

Another limitation is that the Monin–Obukhov profiles applied here may not accurately represent the turbulent flow field of the convective atmospheric boundary layer in reality. Recent studies (Johansson et al. 2001; McNaughton et al. 2007; Cheng et al. 2021) point out that the boundary layer height ${z}_{i}$ is an important length scale characterizing the turbulence flow field but is not included in the Monin–Obukhov similarity. Specifically, the measurements show that the non-dimensional wind velocity gradient depends on ${z}_{i}$ and becomes larger than the Monin–Obukhov profiles (Johansson et al. 2001). Although the non-dimensional temperature gradient shows much less variation with ${z}_{i}$ than the velocity, DNS simulation implies that the temperature profile should be defined by log laws based on ${z}_{i}$, which is at odds with the Monin–Obukhov profile (Cheng et al. 2021). Besides, the horizontal velocity variance also highly depends on ${z}_{i}$ especially when ${z}_{i}$ is close to $L$. As a result, all these factors may induce nonlinear numerical bias from reality when the Monin–Obukhov profile is used to transport the passive scalars. It is necessary to utilize a more accurate flow field to estimate the footprint in the unstable atmospheric boundary layer.

Furthermore, there are also some factors during the adjoint equation simulation that may affect the results of the proposed method, such as the mesh resolution of the domain, the vertical difference $\Delta z$, the numerical scheme for the flux estimation, and the advection scheme for the adjoint concentration, which should be further investigated in the near future.

It should also be stated that it is still debatable as to whether the K–M or FFP method is closer to reality because of the limited number of field-test validations, which are one of the most convincing approaches to validate all models. Kumari et al. (2020) concluded that the K–M model showed smaller errors for different source–receptor deviations based on artificial tracer experiments in unstable conditions over an open field. However, with the tracer experiments at a grassland site, Heidbach et al. (2017) found that the FFP model better predicted the peak contributions of the real footprints, whereas the K–M method led to an overly flattened footprint with an overestimated extent range. Therefore, more field test datasets are necessary for validation, and the proposed method is deemed reliable considering that its results lie between those of the K–M and FFP methods.

## Footprint Simulation of the Urban Canopy

One of the advantages of the proposed method is that heterogeneous dispersion behaviours can be estimated because the complex turbulent flow field used for adjoint simulation can be prepared in advance using CFD techniques. In recent years, more attention has been paid to footprint modelling in urban areas, which is meaningful for gaining insight into atmospheric monitoring that is directly related to large populations. Under these circumstances, the second case employs the proposed method to model the footprint in a block-arrayed urban canopy model.

### Simulation Settings

**Fig. 4:**
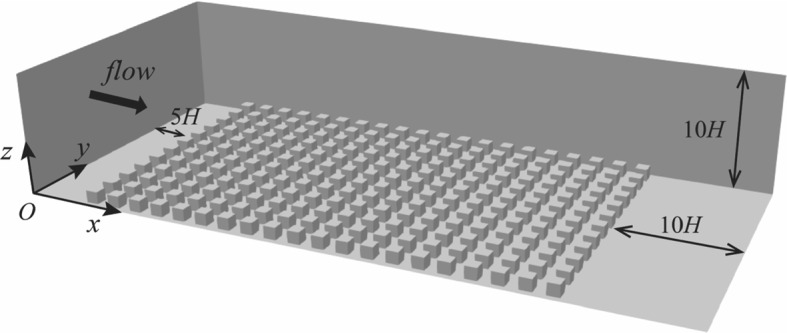
Schematic of the calculation domain for the urban canopy model

The calculation domain is illustrated in Fig. 4. The configuration and size of the blocks follow the settings of an open wind tunnel experiment (WTE) database provided by the Architecture Institute Japan (https://www.aij.or.jp/jpn/publish/cfdguide/), where cubes with edges of $H = 60 \mathrm{mm}$ are uniformly placed at a distance of $H$. This urban building model is a benchmark for exploring the urban wind environment (Xie and Castro 2006; Ikegaya et al. 2017; Wang and Okaze 2022) and pollutant dispersion mechanisms (Kanda and Moriizumi 2009; Tominaga and Stathopoulos 2012) in urban areas.

The size of the domain is set as $54H\left(x\right) \times 24H\left(y\right) \times 10H(z)$ to ensure there is sufficient space to reproduce the turbulent structures around the urban canopy and to observe the footprint distribution over a long distance. The built area is $5H$ away from the inlet and $10H$ away from the outlet for the full development of the wake flow, according to previous research (Tominaga et al. 2008). Regarding the boundary conditions, the inlet employes the same vertical profiles for the velocity, turbulent kinetic energy (TKE), and dissipation as those of the experiment. The outlet is imposed with a zero-gradient boundary for all quantities, except for the pressure, which is set at a fixed value. The side walls have cyclic boundaries, which represent an infinitely wide built area. The top boundary is defined as a slip wall, and the bottom boundary is defined as a nonslip wall with a Spalding wall function.

For the mesh setting, below $z = 1.2H$, a hexahedral orthogonal grid with an edge of $H/16$ is used to resolve the turbulent structures surrounding the buildings. Above $z = 1.2H$, the horizontal size of the grids is maintained, but the vertical edge begins to expand at a ratio of 1.08 to the top boundary. The realizable k–ε RANS model (Shih et al. 1995), which is effective for the dispersion simulation of an urban environment (Tominaga and Stathopoulos 2018), is used to simulate the time-averaged flow field. The Reynolds number based on the freestream wind velocity ${u}_{r}=4.8 \mathrm{m}/\mathrm{s}$ and $H$ is approximately $1.92\times {10}^{4}$.

Regarding the adjoint equation simulation, because the inverse simulation needs to retain all flow field data of the forward simulation and then read it inversely (Jia and Kikumoto 2020), this study conducted a steady simulation of the adjoint equation to reduce the storage cost and only the time-averaged flow field $\overline{u }$ and ${{K }_{t}}$ obtained from forward simulation are required:

20a $$-\left({\varvec{u}}\cdot \nabla \right)\overline{{C }^{*}}-\nabla \cdot \left(\left[{K}_{t}+K\right]\nabla \overline{{C }^{*}}\right)=\delta \left({\varvec{x}}-{{\varvec{x}}}_{m}\right),$$

20b $${K}_{t} = \frac{{\nu }_{t}}{{Sc}_{t}},$$

where ${\nu }_{t}$ is the eddy viscosity estimated by the TKE and dissipation in the RANS model, and ${Sc}_{t}$ is the turbulent Schmidt number, which is set to 0.3 here to offset the underestimation of TKE caused by the RANS model that was proven to be effective in predicting the concentration distribution of passive scalars in the current urban canopy model in a previous study (Lin et al. 2021).

**Fig. 5:**
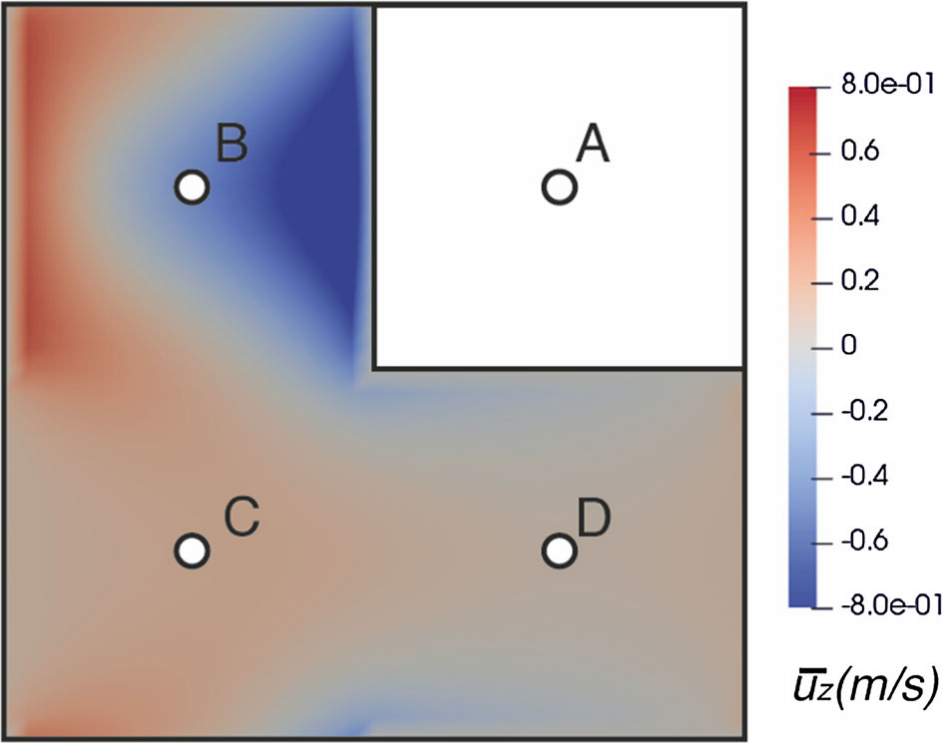
Settings of the sensor positions and distribution of time-averaged vertical velocity ($z=0.3H$)

The footprint modelling is executed for four positions: above the roof (Point A), wake region (Point B), cross-section (Point C), and the road between buildings (Point D), as shown in Fig. 5. Four measurement heights ${z}_{m} = 0.3H$ (except Point A), $1.3H$, $1.8H$, and $2.5H$, are selected to analyze the effects of measurement height on the footprint function. In total, 15 sensors and 45 adjoint equations are simulated for the concentration and flux footprint estimations.

### Simulation Results

#### Validation of the Mean Flow Field

**Fig. 6:**
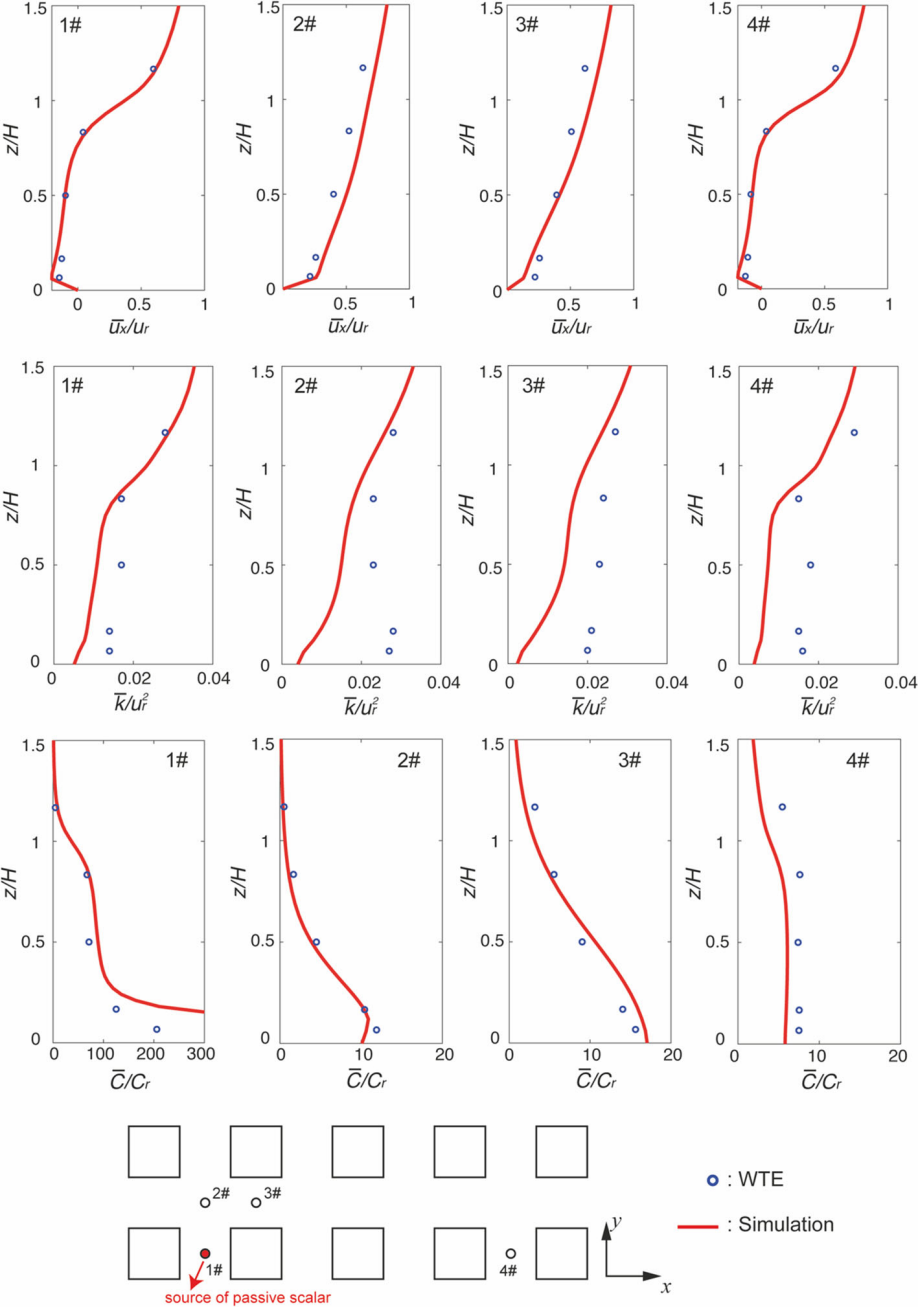
Validation of the streamwise velocity, TKE, and concentration simulated by RANS based on the WTE measurements

Before footprint modelling, it is necessary to validate the CFD simulation against WTE measurements. In addition to the flow field, a forward dispersion simulation is also conducted and validated to discuss the reliability of the dispersion simulation from the side view. The source of the passive scalar is placed at (4*H*, 12*H*, 0). The results of four validation locations are selected and presented in Fig. 6. All results are nondimensionalized by the reference velocity ${u}_{r}$ and the reference concentration ${C}_{r} = {C}_{gas}q/\left({u}_{r}{h}_{r}^{2}\right)$. ${C}_{gas}$ is the scalar concentration of the source, $q$ is the gas flow rate at the source, and ${h}_{r} = 3.33H$ is the reference height.

The validation results of the streamwise velocity are shown in the top row of Fig. 6. The simulated profile agrees well with the measurements, except that the velocity above the block is slightly overestimated at Points 2 and 3. In the middle row, the TKE in the simulation is compared with that of the WTE. In general, although the distribution trend of the profile is similar to the measurement, the RANS model undervalues the TKE, especially in the area below $H$, which is one of the main limitations of RANS simulations of the wind environments surrounding buildings (Tominaga and Stathopoulos 2012). Since ${\nu }_{t}$ is calculated from the TKE in the RANS method, it is worth noting that the influence of this numerical error on the footprint estimations is twofold: 1) ${\nu }_{t}$ is smaller than the true value and suppresses turbulent diffusion during the dispersion simulation, and 2) in Eq. (17), turbulent flux modelling relied on ${K}_{t}$, which may change the distribution of the flux footprint. The bottom row of Fig. 6 shows the validation results of the forward-dispersion simulation. Except for the overestimation of the concentration near the source, the predicted profiles are consistent with the measurements at other locations, which indirectly suggests that the adjoint dispersion also has acceptable accuracy. It is considered that ${Sc}_{t} = 0.3$ effectively mitigates the influence of the underestimated TKE on the dispersion.

After validation, it is necessary to examine the simulated mean vertical velocity $\overline{{u }_{z}}$ at the sensor location because it influences the vertical flux footprint. Occasionally, the local mean flux may change the sign of the footprint values. The $\overline{{u }_{z}}$ of sensors at other heights are close to 0; therefore, only the distribution of $\overline{{u }_{z}}$ at $z=0.3H$ in an elemental area is presented in Fig. 5. Although the overall distribution appears rational in that the effects of the canyon vortex are reflected, the negative region at the windward side of the cube is larger than the LES results (Hellsten et al. 2015; Jia and Kikumoto 2020), which leads to a strong local downwash at Point B and a negative local mean flux. The situations of Points C and D are identical to those of the LES. The canyon vortices that are separated from the cubes escape from the adjacent wake and merge in the open street; thus, $\overline{{u }_{z}}$ at Point C is positive and the $\overline{{u }_{z}}$ at Point D approaches 0.

#### Flux Footprint

It is difficult to evaluate the accuracy of the footprint results estimated by the proposed method because the existing validation dataset for footprints in urban areas is limited. Therefore, we compare the results with a published report (Hellsten et al. 2015) where the footprints are calculated using the LL method for an idealized urban canopy. However, because the results of that study were shown using only colourful contours, and the cube configuration is slightly different from the present research (the distance between cubes was 1.13 $H$ as opposed to $H$), our comparison is discussed qualitatively rather than quantitatively. The flux footprints with two methods are nondimensionalized using their edges of cubes, and the colour bars are unified for comparison.

**Fig. 7:**
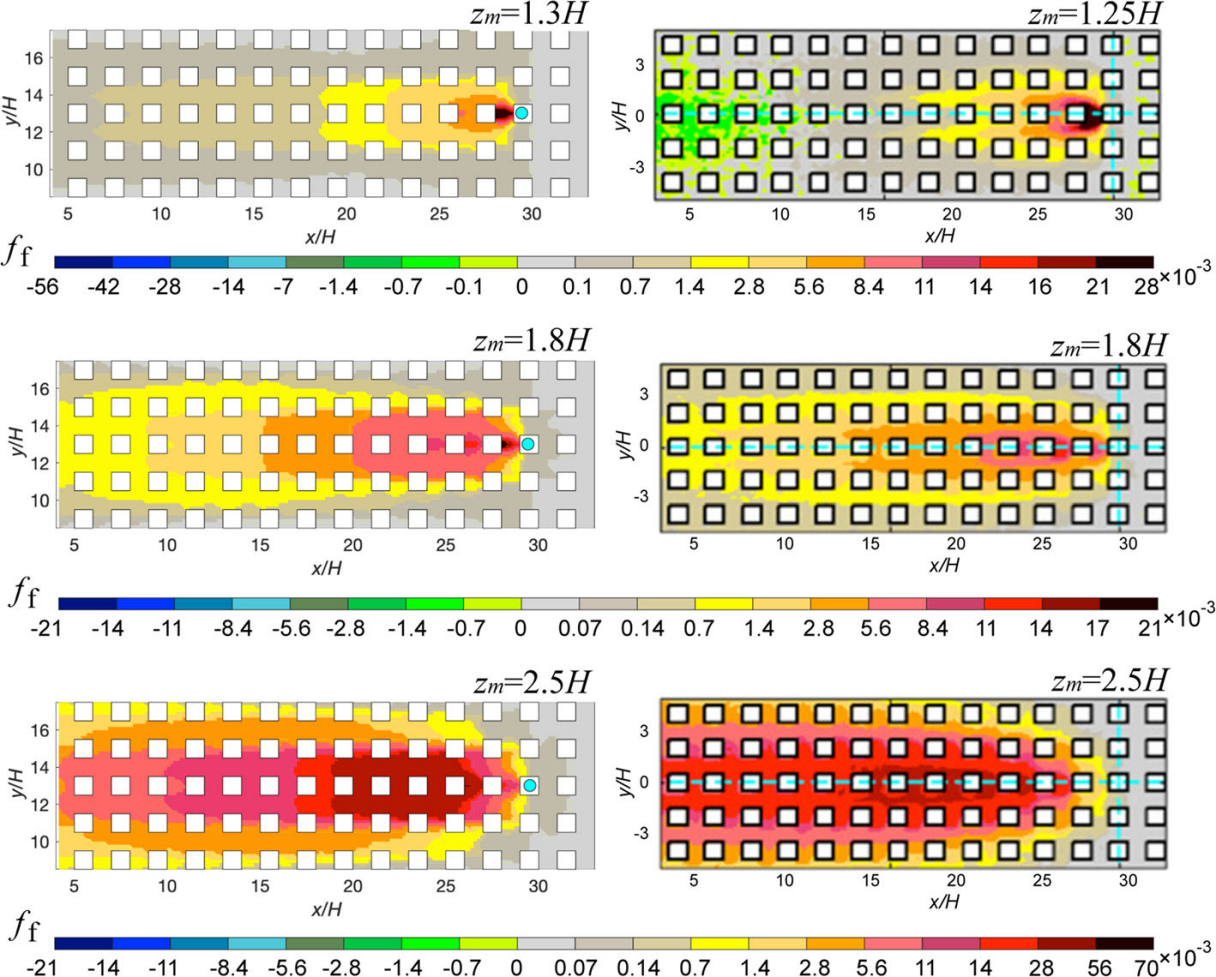
Comparisons of the flux footprints for different measurement heights at Point A estimated by the adjoint method (left) and LL method (right) (Hellsten et al. 2015)

Figure 7 summarizes the flux footprints of the three sensors above the roof (Point A). All three sensors are in the mainstream above the canyon, and the local mean vertical velocity is near 0; therefore, turbulent flux mainly contributed to the footprints. The distributions of the footprints from the adjoint method and LL are similar. With an increase in the measurement heights, the fetch of the footprint expands upstream. The distance between the peak and the sensor increases, whereas the peaks gradually decrease and become flat. Note that the color bars for the three footprints are different to demonstrate the details of the footprints of a large ${z}_{m}$. As described by Hellsten et al. (2015), the shape of the footprint is not the ideal ellipse suggested by the analytical solution (Kormann and Meixner 2001). The footprint distribution is separated by the central row of cubes and has a longer extension along the two streets on the side. This so-called “two-branch” characteristic resulted from the wind field caused by the urban canopy.

When ${z}_{m} = 1.3H$, the peak value is located in the wake region just before the sensor. The spanwise widths of the footprints from the two methods are also consistent and constrained by approximately $5H$. As for the footprints for ${z}_{m} = 1.8H$ and $2.5H$, the peaks are located at the centre line of the sensor and move to the wake regions upstream. The areas surrounding the sensor have little influence, but more source areas in both the streamwise and spanwise directions can be measured at high positions.

The main difference between the results of the two methods at ${z}_{m} = 1.3H$ is that the streamwise range of the LL footprint was shorter than that of the proposed method. In addition, the negative footprint area appeares at approximately $20H$ upstream of the sensor in the LL, which is not observed with the adjoint method. There are two possible reasons for the limitations of the steady-state adjoint equation simulation. First, with the time-averaged flow field produced by RANS, scalars released from upstream areas ascend into the upper stream by the canyon vortex and are transported to the sensor in the streamwise-upward direction, which causes a positive flux. However, when the source areas are $20H$ upstream or further, the scalars move higher than $1.3H$ before they arrive at the sensor location and could not descend in this steady flow. Therefore, the footprint value for the far upstream is near zero instead of negative. In contrast, because LES reproduce most of the turbulence structures and the particles are driven by unsteady flow in the LL, the particles released from the same area could still be transported down to the sensor by turbulent motion, causing negative flux. Second, LES can more effectively predict the turbulent flow field than RANS and can explicitly resolve large-scale turbulent diffusion, which is implicitly modelled by the gradient diffusion hypothesis in RANS. In such an urban canopy model, the adjoint concentration could be overestimated in the streamwise direction and underestimated in the spanwise direction by RANS, because the mean $\overline{{U }_{y}}$ distribution in the wake is opposite along the central line, which prevents spanwise diffusion (Jia and Kikumoto 2021). This causes the streamwise extent of the footprints in the adjoint method to be longer. Therefore, it is necessary to improve the estimation accuracy of the proposed method by implementing LES of the adjoint equation in the future.

**Fig. 8:**
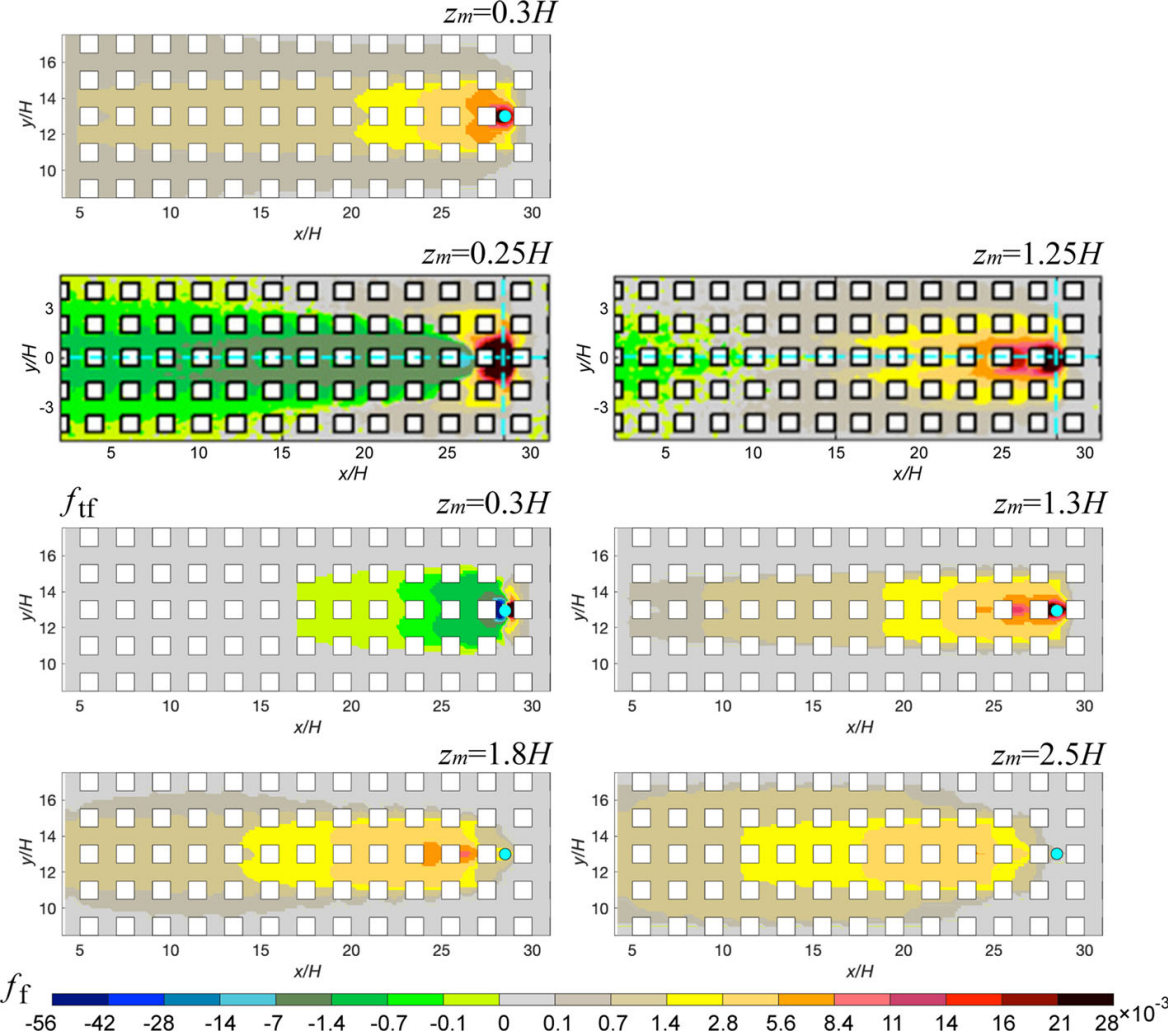
Comparisons of the flux footprints for different measurement heights at Point B estimated by the LL method (second row) (Hellsten et al. 2015) and the adjoint method (remaining rows)

Figure 8 shows the footprints of the sensors located above the wake region (Point B). Hellsten et al. (2015) only includes the results for two heights, as shown in the second row of Fig. 8. When ${z}_{m} = 0.3H$, values in the proposed method are positive, whereas most values in the LL are negative. According to the $\overline{{U }_{z}}$ distribution of RANS in Fig. 5, the sensor is immersed in strongly negative $\overline{{U }_{z}}$. In this case, the resultant mean flux is dominant in the ${F}_{f}$, the vertical fluxes at all positions are predisposed to the negative in Eq. (3), and the footprints become positive during normalization. In contrast, $\overline{{U }_{z}}$ of the sensor is found to be close to 0 in the LES result (Hellsten et al. 2015), so the LL footprints represent the situations of the turbulent flux ${F}_{tf}$.

Considering this, we recalculate the footprint based only on the turbulent flux for ${z}_{m} = 0.3H$, which is shown in the third row of Fig. 8. The result is considerably closer to that with LL. The sensor is immersed in the complicated canyon vortex, where the strong downwash pushes the scalars released from far upstream areas into the wake. Consequently, there are large areas with negative footprint values for both methods. The main difference between the two methods is that the sources surrounding the sensor resulted in more positive effects on the turbulent flux with LL than those with the adjoint method. The negative area begins from the wake side of the sensor in LL, whereas it begins directly at the sensor in the adjoint method, possibly due to the limitation of the mean flow field produced by the RANS model. In such a steady flow field, the particles released from the source areas downstream of the sensor could reach the sensor only by the backward-upward motion of the canyon vortex, which naturally leads to a positive vertical flux. Meanwhile, most of the particles released from the upstream areas are transported downward into the wake from the mainstream, causing the vertical flux to become negative. In the unsteady flow of the LES, the particles released from the same wake region as the sensor can still move upward to the sensor and cause a positive vertical flux.

For the measurement heights ${z}_{m} = 1.3H$, $1.8H$, and $2.5H$, the situation is similar to that at Point A. The peak of the footprint distribution becomes farther from the sensor and flatter with increasing ${z}_{m}$; The “two-branch” characteristic persists. Moreover, the crosswind range of the footprint becomes wider for higher sensors.

**Fig. 9:**
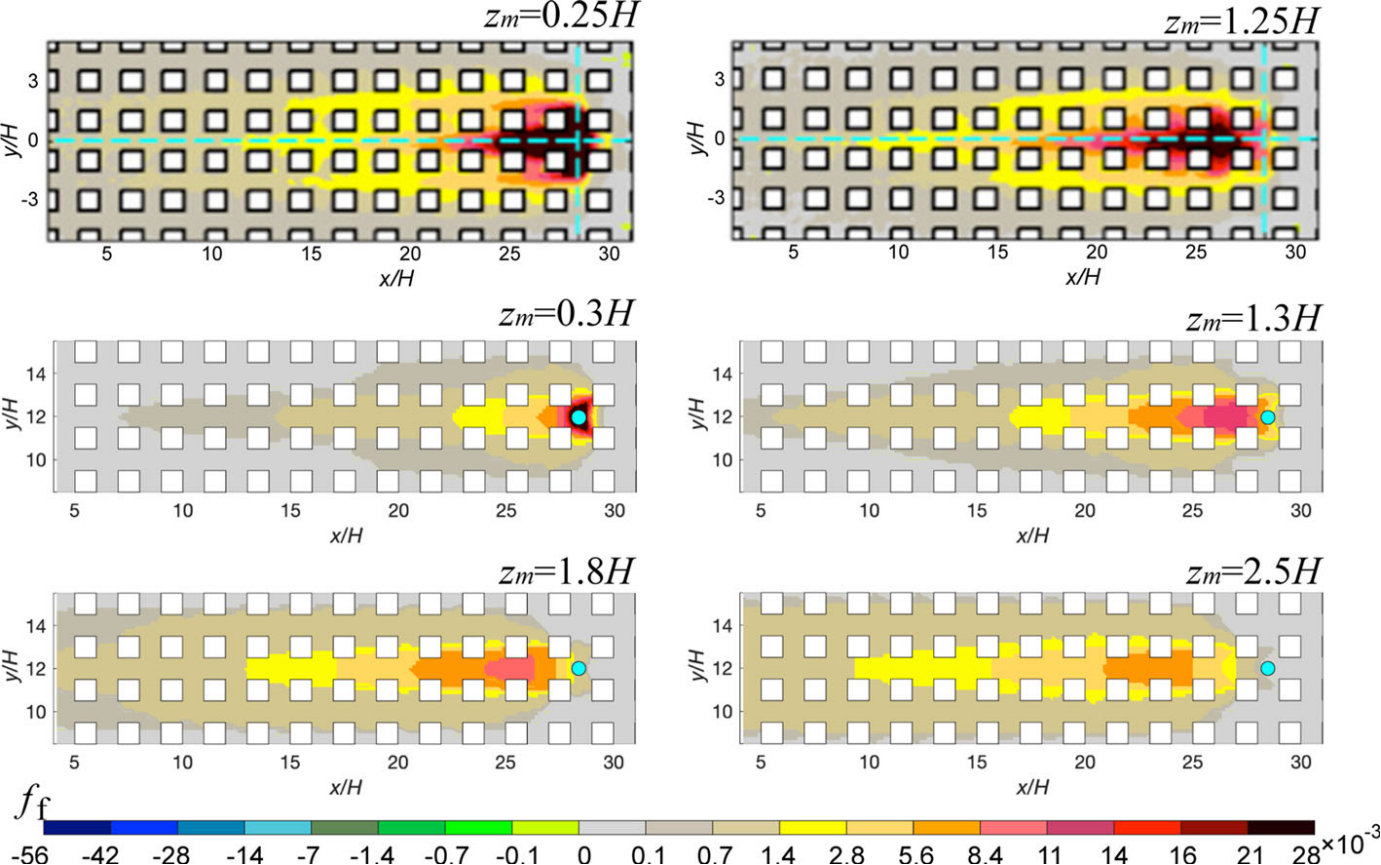
Comparisons of the flux footprints for different measurement heights at Point C estimated by the LL method (first row) (Hellsten et al. 2015) and the adjoint method (remaining rows)

Point C is located at the cross section of the cubes. Hellsten et al. (2015) only provides the LL results for two heights; therefore, the footprints for ${z}_{m} = 0.3H$ and $1.3H$ are compared in Fig. 9. At both heights, the majority of the footprints concentrates on the central street where the sensor is located. At ${z}_{m} = 0.3H$, except the central street, large footprint values can also be observed in two adjacent streets, which is called the “three-branch” pattern in Hellsten et al. (2015). However, the crosswind dispersion is again insufficient in RANS when compared to LL due to the previously discussed reason. Hence, the “three-branch” pattern only remains in the central branch with the adjoint method. Nonetheless, the footprints at ${z}_{m} = 1.3H$ are similar.

**Fig. 10:**
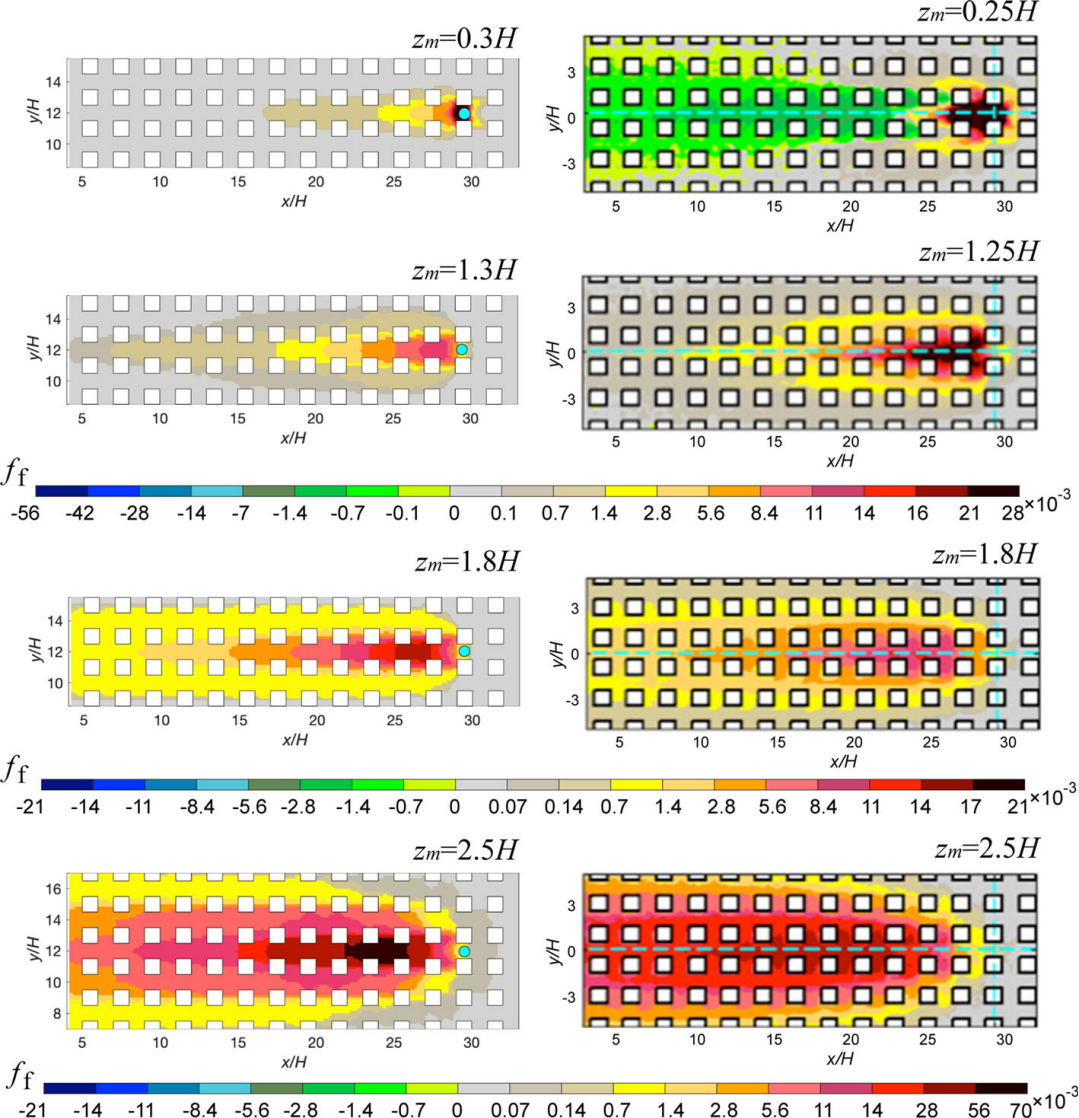
Comparisons of the flux footprints of different measurement heights at Point C estimated by the adjoint method (left column) and the LL method (Hellsten et al. 2015) (right column)

The footprints of Point D are shown in Fig. 10. For the sensor below the canopy height, the footprint estimated by the adjoint method has a longer positive region and shows no negative region compared to that of LL. The mean vertical velocity in the open street is slightly positive, as shown in Fig. 5, because the vortices from the adjacent canyons are separated by the cube and then merge. Under these circumstances, scalars released from far upstream sources climb up by the mean flow before they reach the sensor. However, in the LL, although the released particles are also driven upward by the local-mean flow, the strong turbulent motions near the sensor may have forced them downward, resulting in a negative flux. In addition, the proposed method again fails to predict three branches in the LL results because of the limited dispersion of the adjoint concentration in the spanwise direction, as discussed above. The situation for ${z}_{m} = 1.3H$ is similar to that for Point C. When ${z}_{m}$ changes to $1.8H$ and $2.5H$, the footprints still concentrate on the central open street and become larger and flatter.

When the footprints of the four points at the same height across Figs. 7, 8, 9 and 10 are compared, the low measurement height corresponds to strong horizontal heterogeneity. The shape of the footprints appears to depend on the sensor positions, especially for ${z}_{m}=0.3H$ and $1.3H$. When the sensors are elevated, the footprints become similar, which means that they gradually become independent of the skewed flow field caused by the urban canopy. They can also be divided into two groups based on their analogies. The footprints in each group are almost identical, especially when ${z}_{m}$ is greater than $1.5H$. Point A and B are placed in Group I, which is in the row of cubes. Their footprints are separated into two branches by the central row of the cubes. Points C and D are in Group II, which is in the row of the open street. Their footprints are concentrated on the open street where the sensors are located.

#### Concentration Footprint

**Fig. 11:**
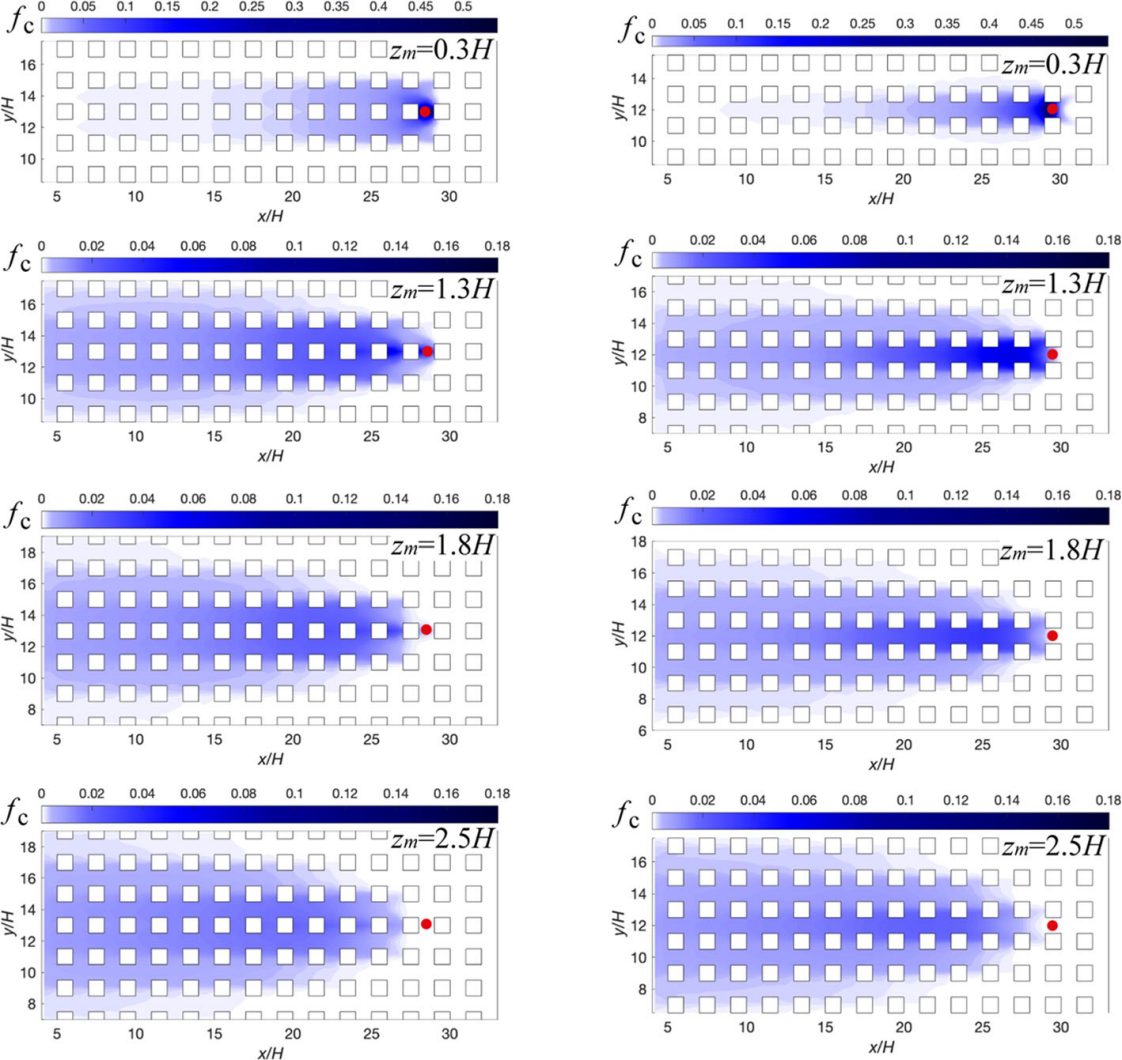
Concentration footprints for different measurement heights at Point B (left column) and Point D (right column) estimated by the adjoint method

In this section, the concentration footprints estimated using the proposed method are discussed. As described in Sect. 2, the concentration footprint can be directly obtained by the adjoint concentration released from each sensor. The footprints of the two groups described in Sect. 4.2.2 are comparable for different sensor positions; therefore, we only show the results of Points B and D in Fig. 11.

The characteristics of the concentration footprints are analogous to those of the flux footprints. With increasing measurement heights, the footprints become longer and wider, with smaller peak values and flatter shapes. The passive scalars tend to move upward after release and generally did not return to the canyon; therefore, the sensor located below the cube height only covered limited source areas. In contrast, higher sensors could receive considerably more effects from a source far upstream. The footprint distribution of Point B has a two-branch feature that is separated by the central row of cubes, whereas the footprint distribution of Point D has a one-branch feature that developed along the open street. The horizontal difference caused by the sensor position gradually disappears with the increase in the measurement heights.

In general, the concentration footprint estimated by the adjoint method is qualitatively comparable to the results of LL, whereby the main features of the footprints in the urban canopy model are well reproduced.

## Conclusions

This study proposed a backward Eulerian footprint modelling approach based on the application of the adjoint equation. The concentration footprint can be conveniently obtained by the adjoint concentration distribution, and the flux footprint can be computed from the gradient of the adjoint concentration field using K-theory. The proposed method was first evaluated in footprint modelling for three-dimensional ABLs with different stability conditions against the K–M model and FFP models. The results were reasonable in that the proposed method performed similarly to the FFP method in terms of convective stability, and to the K–M method in terms of stable stability because of the assumptions regarding turbulent diffusion.

To understand the ability and practicality of the proposed method, it was applied to estimate the footprints in a block-arrayed urban canopy based on the time-averaged flow field simulated by RANS. The results were qualitatively compared to those in the existing literature using the LL method. The proposed method succeeded in predicting the main features of footprints caused by the urban canopy terrain with different measurement positions and heights. Footprints of the sensors in the same row of cubes were separated into two branches, whereas footprints of the sensors located in the open-street region dominantly developed upstream along the street, which clearly differed from the ideal ellipse distribution yielded by the analytical solution. Additionally, the numerical errors caused by the steady simulation of the adjoint equation using RANS were discussed. The imperfect predictions of the mean vertical velocity field in the wake region and insufficient modelling of the turbulent diffusion affected the reliability of footprint modelling for the sensors in the canyon.

There are some limitations to the proposed method. First, the turbulent flux was estimated by the vertical gradient of the local concentration, which may lose credibility when the diffusive process is more significant than the advective process (Sogachev and Lloyd 2004) or when the turbulent diffusivity is highly anisotropic. It is necessary to improve turbulent flux estimation using a higher-order closure model. In addition, according to Eq. (13), the gradient estimation depends on the vertical difference $\Delta z$. The numerical stability against the scale of $\Delta z$ should be investigated further. Moreover, the performance of the proposed method depends on an accurate simulation of the flow field in the target domain. The application of the Monin–Obukhov profiles in the first case and RANS in the second case both resulted in numerical flaws to some extent. For complex-built areas, LES could better simulate the turbulent flow fields and conduct an unsteady simulation of the adjoint equation. This option can explicitly resolve turbulent structures with different scales, and the turbulent flux can be directly captured through the time-series of the adjoint concentration and velocities, which solves the problem. However, the heavy calculation burden and large amount of inverse simulation data with LES remain a challenge. Finally, the proposed method was validated by quantitative comparisons to the K–M and FFP models in the first case, and qualitative comparison with the LL method in the second case, owing to the lack of a validation database based on experiments or field tests. To evaluate existing footprint approaches thoroughly, it is critical to construct these validation databases in the future.
